# Delayed onset porous polyethylene implant-related inflammation after orbital blowout fracture repair: four case reports

**DOI:** 10.1186/s12886-016-0287-0

**Published:** 2016-07-07

**Authors:** Orapan Aryasit, Danny S. Ng, Alice S. C. Goh, Kyung In Woo, Yoon-Duck Kim

**Affiliations:** Department of Ophthalmology, Faculty of Medicine, Prince of Songkla University, Hat Yai, Songkhla Thailand; Department of Ophthalmology and Visual Sciences, Faculty of Medicine, The Chinese University of Hong Kong, Kowloon, Hong Kong; International Specialist Eye Center (ISEC), Kuala Lumpur, Malaysia; Samsung Medical Center, Sungkyunkwan University School of Medicine, Seoul, South Korea

**Keywords:** Delayed inflammation, Orbital fracture, Porous polyethylene

## Abstract

**Background:**

Porous polyethylene implants are commonly used in orbital blowout fracture repair because of purported biocompatibility, durability, and low frequency of complications. Delayed inflammation related to porous polyethylene sheet implants is very rare and no case series of this condition have been reported.

**Case Presentation:**

This is a retrospective review of clinical presentations, radiographic findings, histopathological findings, treatments, and outcomes of patients who developed delayed complications in orbital blowout fracture repair using porous polyethylene sheets. Four male patients were included with a mean age of 49 years (range 35–69 years). Blowout fracture repair was complicated with implant-related inflammation 10 months, 2 years, 3 years, and 8 years after surgery. Chronic and subacute orbital inflammatory signs were noted in two patients and acute fulminant orbital inflammation was found in two patients. Three patients developed peri-implant abscesses and one patient had a soft tissue mass around the implant. All patients underwent implant removal and two of these patients with paranasal sinusitis had sinus surgery. Histopathological findings revealed chronic inflammatory changes with fibrosis, and one patient had foreign body granuloma with culture positive *Staphylococcus aureus*.

**Conclusions:**

Delayed complications with porous polyethylene sheets used in orbital blowout fracture repair may occur many years following the initial surgery in immunocompetent patients. Low-grade or fulminant inflammation could complicate blowout fracture repair related with the implant.

## Background

Delayed complications related to alloplastic implant materials in orbital blowout fracture repairs are infrequent and generally appear as isolated case reports. Warrier et al. reported inflammation and infection that developed 1.5–20 years after silicone implants for orbital fracture repair [1]. Custer et al. described six cases of late infection/inflammation in supramid implants ranging from 8 to 16 years after implantation [2]. Long-term durability and safety of porous polyethylene implants for orbital fracture reconstruction have been reported [3, 4]; however, there have been very few reported late complications. In a retrospective review of 30 patients, Ng et al. reported a patient who developed delayed onset recurrent implant infection leading to implant removal [5]. Samimi et al. reviewed 21 explanted periorbital biomaterials due to nonresolving infection or exposure and reported one immunosuppressed patient who had granulomatosis with polyangiitis with an infected porous polyethylene sheet after 3 years [6]. The former case developed several bouts of inflammation from 6 months after fracture surgery, and finally had the implant removed 36 months after the original surgery [5]. In addition, the latter case involved an immunocompromised patient [6].

In this case series, we describe the clinicopathological features of inflammation after orbital blowout fracture repair using porous polyethylene sheets, which is rarely encountered after a long postoperative duration in immunocompetent patients [7].

## Case presentation

Medical records of four consecutive patients who developed delayed complications related with porous polyethylene sheets after orbital blowout fracture repair at Samsung Medical Center between 2007 and 2010 were retrospectively reviewed. Delayed onset inflammation was regarded if an implant-related inflammation occurred 6 months later than the fracture repair. The clinical presentations, radiographic findings, histopathological findings, and treatment and outcome data were collected. The Samsung Medical Center Institutional Review Board approved the retrospective review of the patients’ data, and the study adhered to the tenets of the Declaration of Helsinki.

All patients were male with mean age of 49 years (range 35–69 years) (Table 1). None had a significant past medical history or current disease. Orbital blowout fracture occurred after automobile traffic accidents (two patients), sports injury (one patient), and an accidental fall (one patient). All patients had uneventful blowout fracture repair using porous polyethylene sheets (Medpor®, Porex Surgical, Newnan, GA, USA) of 1 mm thickness for correction of enophthalmos and diplopia, using a transconjunctival approach for the inferior wall and a transcaruncular approach for the medial wall fracture. All implants were soaked in gentamicin solution before implantation. Methylprednisolone (250 mg) was infused intravenously at the end of the surgery, and postoperative systemic antibiotics were administered. None of the patients showed sinusitis at the time of blowout fracture repair.

**Table 1:** Clinical presentations, radiographic findings, histopathological findings, treatments, and outcomes

Patient number/Duration of onset of complication/Location of implant	Age (yrs^a^)	Clinical presentation	Imaging studies	Histopathological findings/Results of culture	Treatments	Outcomes	F/U^b^ time (mos^c^)
1/8 years/Medial	41	Eye pain, diplopia with 2 mm hyperglobus, hypoesthesia for 14 days	Peri-implant soft tissue mass, clear sinus in CT^e^	Fibrosis with chronic inflammation and calcification/negative culture	Implant removal, mass debulking	Postoperative persistent hypoesthesia of the cheek, 0.5 mm of the hyperglobus	4.7
2/3 years/Inferomedial	69	Persistent eyelid swelling for 5 months	Peri-implant low-signal intensity with surrounding tissue enhancement in T1-enhanced MR^f^, clear sinus	Foreign body granuloma/positive CoNS^d^ culture	Implant removal, abscess drainage	Infection resolved without complications	3.1
3/2 years/Medial	51	Eye pain, skin redness, conjunctival injection, eyelid swelling, hyperdeviation with diplopia for 2 days	D-shaped low density mass with enhanced rim, mucosal thickening of ethmoid and maxillary sinuses in CT	Chronic sinusitis with eosinophils, features compatible with inflammation in the nasal mucosa/negative culture	Implant removal, abscess drainage, sinus drainage by ENT^g^ surgeons	3 mm of enophthalmos	5.1
4/10 month/Inferior	35	Eye pain, skin redness, conjunctival injection, eyelid swelling, proptosis for 1 day	D-shaped soft tissue-density mass with enhanced rim, mucosal thickening of ethmoid and maxillary sinuses in CT	Chronic inflammation in the nasal mucosal tissue/negative culture	Implant removal, abscess drainage, sinus drainage by ENT surgeons	Infection resolved without complications	2.1

^a^ *Yrs* years, ^b^ *F/U* follow-up, ^c^ *mos* months, ^d^ *CoNS* coagulase negative *Staphylococcus aureus*, ^e^ *CT* computed tomography, ^f^ *MR* magnetic resonance imaging, ^g^ *ENT* ear, nose and throat

**Fig. 1:**
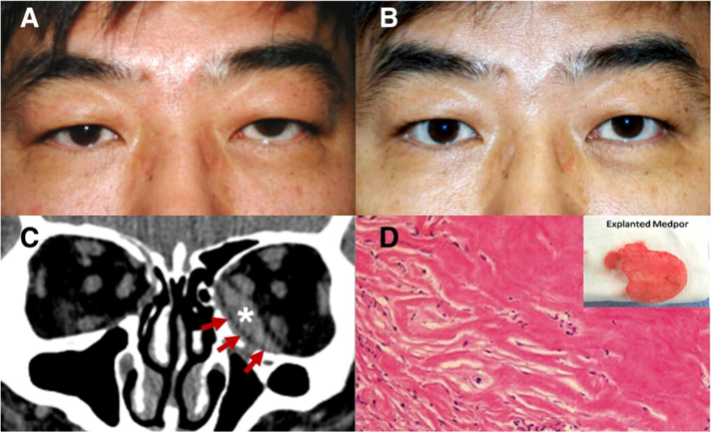
Patient 1 **a** A 41-year-old male presented with eye pain, diplopia with 2 mm hyperglobus and hypesthesia for 14 days. **b** Four months after explantation. **c** Coronal CT showed a soft tissue mass (asterisk) around the radiolucent sheet (arrows). **d** The histopathological finding revealed fibrosis with chronic inflammatory cell infiltration (inset: explanted implant) (hematoxylin and eosin staining, 200×)

**Fig. 2:**
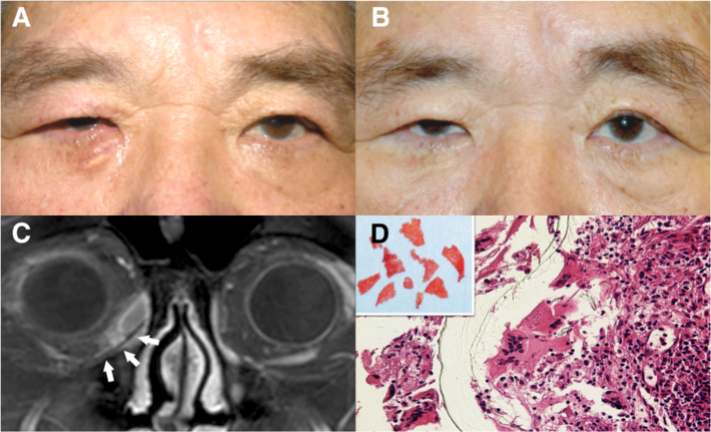
Patient 2. **a** A 69-year-old male presented with persistent swelling at the inferomedial side of the right eye for 5 months. **b** Three months after explantation. **c** Coronal magnetic resonance (MR) imaging showed peri-implant low signal intensity (arrows) with high signal intensity in the surrounding tissue at the inferomedial orbit in the gadolinium-enhanced fat-saturated T1 image. **d** The histopathological findings showed foreign body granuloma with inflammatory cell aggregation adjacent to the fragmented polyethylene sheet (inset: explanted implant) (hematoxylin and eosin staining, 400×)

The onset of symptoms varied among the patients. Patients 1 and 2 presented with subacute and chronic eyelid swelling and pain, respectively (Figs. 1a, 2a). Acute fulminant orbital inflammation was seen in patients 3 and 4, who had eye pain, conjunctival injection, and eyelid swelling with concurrent sinusitis. Patient 4 had an upper respiratory infection 2 weeks prior to the presentation of orbital inflammation. None of them had any anterior and posterior segment abnormalities or optic nerve dysfunction. The implants for the patients were well placed in the computed tomography (CT) scan except for one patient. The implant in the patient 3 was misplaced in the posterior orbit showing a gap between bony edge and the implant. All four patients underwent surgical exploration and implant removal under coverage of systemic antibiotics. The fibrotic tissue around the implant was resected partially and left behind for avoidance of vital orbital tissue damage. All the explanted implants were submitted for Gram staining and microbial culture.

Histopathological findings of the orbital mass revealed fibrosis and chronic inflammation (Fig. 1). Patient 2 showed foreign body granuloma with giant cell infiltration adjacent to the fragmented implant spicules (Fig. 2). Coagulase negative *Staphylococcus aureus* was cultured from the explant. All patients had clinical resolution after explantation and systemic broad spectrum antibiotic treatment (Figs. 1b, 2b).

## Discussion

Delayed inflammation related to orbital implantation for orbital fracture repair is very rare. Approximately 350 cases underwent orbital fracture repair using porous polyethylene sheets during the same period from 2007 to 2010 at Samsung Medical Center. Furthermore, there were no other cases out of 1000 patients throughout the entire surgical log of orbital fracture repair at the same institution since 1994. This complication is very rare but should be reported for clinicians who care for orbital fracture patients.

**Fig. 3:**
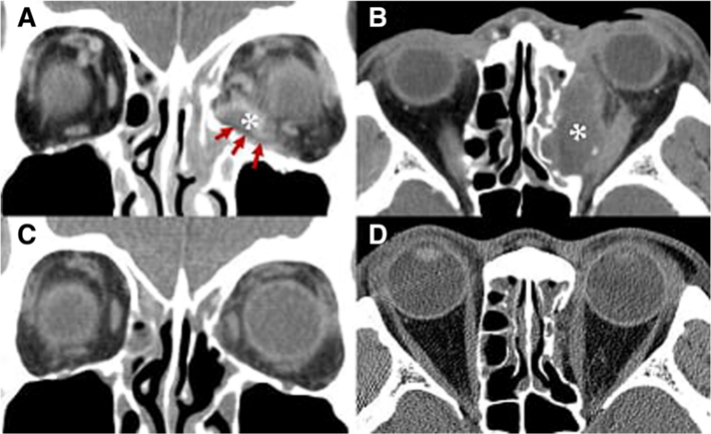
Patient 3. **a** Coronal CT showed a D-shaped low density mass (asterisk) adjacent to the radiolucent sheet (arrows). Sinus opacification was evident in the frontal and ethmoid sinuses. **b** An axial CT image showing a large low density mass (asterisk) extending to the entire medial wall of the orbit. **c, d** Five months after the explantation and sinus surgery

A lack or reduction of fibrovascularization into the implant for orbital fracture repair might play a role in implant infection. Porous polyethylene is susceptible to infection in the early postoperative period before sufficient fibrovascular ingrowth occurs in 3 months [3, 8–11]. Our patient series showed delayed onset of porous polyethylene sheet-related infection or inflammation after 10 months and up to 8 years, which lagged beyond the duration for fibrovascularization. Mauriello et al. studied 10 patients who developed infections after alloplastic implants with silicone and gelatin film for orbital floor fracture repair, and noted that the predisposing factors were dental surgery, upper respiratory infection, implant extrusion into the maxillary sinus, medial implant migration resulting in dacryocystitis, rhinoplasty, and snorting cocaine [12]. Custer et al. reported small fistulous tracts between the supramid implant capsule and the maxillary sinus that led to infection [2]. In our case series, we speculate that the implanted porous polyethylene sheet and integrated surrounding fibrous tissue adjacent to the paranasal sinuses could still be an incompetent barrier to sinus infection, even after a long postoperative period. In patient 3, the edge of the implant did not cover the whole defect of the medial wall fracture, and which might serve as a precipitating cause of infection (Fig. 3).

Patient 2 showed chronic inflammatory signs with abscess formation without sinusitis. The explanted porous polyethylene sheet was brittle and histopathological examination showed foreign body granuloma adjacent to the implant spicules. Microbial infection and long-term tissue inflammation could result in implant degradation. In an experimental study to examine the responses of implanted porous polyethylene after direct inoculation of *Staphylococcus aureus* into rats, electron microscopy showed bacteria and active inflammatory infiltrates on the degraded implant surface [11]. In another animal study, giant cells were detected at the interface between the implants and surrounding granulation tissue, indicating a chronic foreign body reaction [13]. In specific circumstances, porous polyethylene in the fracture site can precipitate chronic inflammation and foreign body reactions.

Three of the patients in this series were culture negative for microorganisms. We could not determine whether the reasons involved prior use of antibiotics or sterile inflammation.

Absorbable alloplastic materials are manufactured and used for orbital wall fracture. They were originally designed to sustain the prolapsed orbital tissue as long as the implant support was needed, and not to serve as a foreign body in the fracture site [14–20]. Long-term follow-up and accumulation of clinical experiences can help identify the proper implant for orbital wall fracture repair.

## Conclusions

Porous polyethylene implants are commonly used in orbital blowout fracture repair because of purported biocompatibility, durability, and low frequency of complications. However, delayed onset of porous polyethylene implant infection or inflammation may complicate orbital fracture repair. Porous polyethylene sheets may provide an incompetent barrier to sinus infection, and can remain as a foreign body in the fracture site, resulting in an implant-related inflammation.

## Abbreviations

CT, computed tomography; ENT, ear, nose and throat; MR, magnetic resonance
